# Genome-Wide Investigation of the Auxin Response Factor Gene Family in Tartary Buckwheat (*Fagopyrum tataricum*)

**DOI:** 10.3390/ijms19113526

**Published:** 2018-11-09

**Authors:** Moyang Liu, Zhaotang Ma, Anhu Wang, Tianrun Zheng, Li Huang, Wenjun Sun, Yanjun Zhang, Weiqiong Jin, Junyi Zhan, Yuntao Cai, Yujia Tang, Qi Wu, Zizhong Tang, Tongliang Bu, Chenglei Li, Hui Chen

**Affiliations:** 1 College of Life Science, Sichuan Agricultural University, Ya’an 625014, China; lmyyunxi@163.com (M.L.); WaohUncle_Ma@163.com (Z.M.); kobezey@163.com (T.Z.); 18428385443@163.com (L.H.); sunnan82475@163.com (W.S.); 15227793373@163.com (Y.Z.); Joan541573@163.com (W.J.); zhanjunyi0412@163.com (J.Z.); caiyt410725@163.com (Y.C.); 15984549383@163.com (Y.T.); wuqi@sicau.edu.cn (Q.W.); 3530279123456789@163.com (Z.T.); tlbu@163.com (T.B.); lichenglei1998@163.com (C.L.); 2 College of Agricultural Science, Xichang University, Xichang 615000, China; 13795660264@163.com

**Keywords:** tartary buckwheat, ARFs, genome-wide, fruit development, expression patterns

## Abstract

Auxin signaling plays an important role in plant growth and development. It responds to various developmental and environmental events, such as embryogenesis, organogenesis, shoot elongation, tropical growth, lateral root formation, flower and fruit development, tissue and organ architecture, and vascular differentiation. However, there has been little research on the *Auxin Response Factor* (*ARF*) genes of tartary buckwheat (*Fagopyrum tataricum*), an important edible and medicinal crop. The recent publication of the whole-genome sequence of tartary buckwheat enables us to study the tissue and expression profile of the *FtARF* gene on a genome-wide basis. In this study, 20 *ARF* (*FtARF*) genes were identified and renamed according to the chromosomal distribution of the *FtARF* genes. The results showed that the *FtARF* genes belonged to the related sister pair, and the chromosomal map showed that the duplication of *FtARFs* was related to the duplication of the chromosome blocks. The duplication of some *FtARF* genes shows conserved intron/exon structure, which is different from other genes, suggesting that the function of these genes may be diverse. Real-time quantitative PCR analysis exhibited distinct expression patterns of *FtARF* genes in various tissues and in response to exogenous auxin during fruit development. In this study, 20 *FtARF* genes were identified, and the structure, evolution, and expression patterns of the proteins were studied. This systematic analysis laid a foundation for the further study of the functional characteristics of the *ARF* genes and for the improvement of tartary buckwheat crops.

## 1. Introduction

Auxin signaling plays an important role in plant growth and development. It responds to various developmental and environmental events, such as embryogenesis, organogenesis, shoot elongation, tropical growth, lateral root formation, flower and fruit development, tissue and organ configuration, and vascular differentiation [1]. At the molecular level, most of these processes are controlled by auxin response genes [2,3], and auxin coordinates plant development by regulating expression of auxin/indole-3-acetic acid (Aux/IAA), Gretchen Hagen 3 (GH3), and small auxin-up RNA (SAUR) [4,5]. The response to auxin is generated by conserved promoter elements, including TGA-elements, AuxRR-core, and AuxRE. Auxin promoters are activated by plant-specific transcription factors designated auxin response factors (ARFs) [6,7]. Most ARFs contain a highly conserved N-terminal B3 DNA-binding domain (DBD) that includes an ARF family-specific domain and recognizes AuxRE in the auxin response gene promoter [8] and a carboxyl-terminal dimer domain (CTD), similar to that of Aux/IAAs C, which is a protein–protein interaction domain that mediates the homodimerisation and heterodimerization of ARFs, as well as the heterodimerization of the ARF and Aux/IAA proteins [9,10,11,12]. The intermediate region (MR) between DBD and CTD activates or suppresses transcription according to its amino acid composition [8].

It is reported that the ARF proteins are encoded by a large family and are conserved throughout the evolution of the plant kingdom, and since the expansion of the family seems to be related to the evolution and diversity of plants, the function of the ARFs has been thoroughly studied [13,14]. ARF1 and ARF2 loss-of-function mutants in Arabidopsis thaliana affected leaf senescence and floral organ exfoliation [15]. The functionally deficient ARF3 mutant showed defects in pistil and flower meristem [16,17]. AtARF7 was involved in the conditional regulation of the differential growth of aerial tissues, while the absence of AtARF7 weakened the response of the hypocotyls to blue light and auxin stimulation [18]. AtARF8 regulated hypocotyl elongation, auxin homeostasis, and fruit development [15,19]. In addition, the flowers of the double mutants of ARF6/ARF8 were sterile buds with short petals, short stamens, and unsplit anthers [20]. The ARF7/ARF19 double mutation affects auxin-mediated lateral root development [21]. Transgenic rice plants (*Oryza sativa* L.) expressing antisense *OsARF1* were very slow to grow and leaves were curly and were barren, indicating that this gene is very important to the nutritional and reproductive development of rice [22]. In tomato (*Solanum lycopersicon*), recent studies have shown that the *SLARF* genes are involved in flower development and fruit formation, development, and maturation [23,24]. The *ARF* gene family evolved to sense how auxin signals evolve as plants move from single cells to more complex multicellular flowering plants. The existence of more and more sequenced genomes has facilitated the evolutionary studies of the *ARF* gene family and enabled people to conduct large-scale analysis of the *ARF* genes in many different species, which would be helpful to understand their evolutionary origin and biological functions.

Tartary buckwheat (*Fagopyrum tataricum*) is a type of cultivated medicinal and edible crop with good economic and nutritional value whose essential amino acid composition of the seed protein is balanced, and the total content is also higher than that of the primary grain crops [25,26]. Although ARFs affect root tip dominance, vascular development, tropical movement, root growth, tissue and organ morphology, and flower and fruit development [27], many problems remain unresolved. The *ARF* gene family has been widely studied in many plants. FtARF2 plays a potential role in the final fruit size of tartary buckwheat [28]. However, the basic knowledge of the ARF protein in tartary buckwheat is limited. Because of the importance of the *ARF* gene in various physiological processes, it is worthwhile to systematically study the FtARF family. The recently completed genome sequencing of tartary buckwheat provides an opportunity to reveal the tissue, expression, and evolutionary characteristics of the *FtARF* gene family at the whole genome level [29]. In this study, we provide detailed information on the exon–intron organization, motif compositions, genomic structures, chromosomal locations, sequence homology synteny, and expression patterns of 20 *tFtARF* genes. In addition, the phylogenetic relationship between the *ARF* genes in Arabidopsis, rice, maize, and tartary buckwheat was also compared. Global expression analysis was performed to identify the involvement of specific *FtARF* gene family members in different biological processes. In particular, the participation of *FtARF* genes in tartary buckwheat fruit development was studied in detail, and the specific *FtARF* genes were screened out by using exogenous auxin. This study provided valuable insights for the functional characterization of *ARF* gene family members in tartary buckwheat growth and development.

## 2. Results

### 2.1. Identification of FtARF Genes

To identify the *FtARF* genes, two Basic Local Alignment Search Tool (BLAST) methods were used to identify all possible ARF members in the tartary buckwheat genome. Through these two methods, more than 36 *ARF* genes were identified from the tartary buckwheat genome. Because buckwheat genomes are sequenced using a genome-wide shotgun strategy, some of these *ARF* genes may be redundant, although they are located on different scaffolds. A total of 20 potential ARF proteins were identified to be associated with the *FtARF* gene by removing the redundant and alternate forms of the same gene (Table S1). In this study, the nomenclature system of FtARF temporarily uses FtARF1 to FtARF19 (with the exception of FtARF7, FtARF10, FtARF12, FtARF14, FtARF15, and FtARF18) to differentiate each *ARF* gene from the AtARF homology with *FtARF* genes. Seven other homologous genes were not found in Arabidopsis thaliana, so it is not possible to name them according to the homologous proteins in Arabidopsis thaliana. We named them based on the homology of the seven genes to the *ARF* genes of rice and maize.

Gene characteristics included coding sequence length (CDS), predicted protein molecular weight (MW), isoelectric point (pI), and subcellular localization. Of the 20 FtARF proteins, FtARF13 was the smallest protein with 331 amino acids (aa), and the largest one was FtARF19 (1083 aa) (Table S1). The MW of the proteins ranged from 37.2 to 120 KDa, and the pI ranged from 5.34 (FtARF5) to 8.30 (FtARF15). The predicted subcellular localization results showed that 18 FtARF proteins were located in the nuclear region, while 2 FtARF proteins were located in the chloroplast.

### 2.2. Multiple Sequence Alignment, Phylogenetic Analysis, and Classification of FtARF Genes

**Figure 1:**
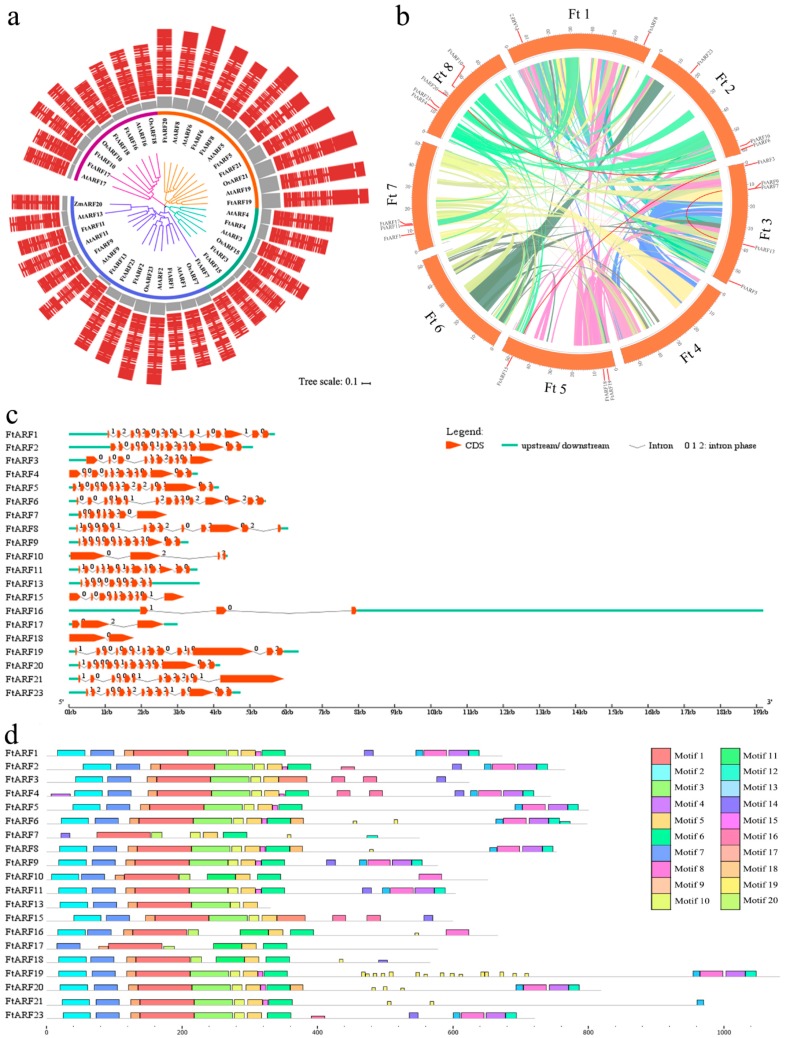
*ARF* gene family in tartary buckwheat. (**a**) Unrooted phylogenetic tree representing relationships among the FtARFs. The different-colored arcs indicate different groups. Gray columns represent protein lengths. Red columns represent protein sequence structure. (**b**) Schematic representations of the chromosomal distribution and interchromosomal relationships of the *FtARF* genes. Colorized lines indicate all synteny blocks in the tartary buckwheat genome, and the red lines indicate duplicated *ARF* gene pairs. The chromosome number is indicated at the bottom of each chromosome. (**c**) Exon–intron structure of *FtARF* genes. Orange boxes indicate exons; green lines indicate introns. The number indicates the intron phase. (**d**) The motif composition of FtARF proteins. The motifs, numbered 1–20, are displayed in different-colored boxes. The sequence information for each motif is provided in Table S2. The length of protein can be estimated using the scale at the bottom.

The phylogenetic relationship of the FtARF proteins was studied by multiple sequence alignment of the ARF domain involving approximately 81 amino acids. The sequences of the ARF domains were highly conserved as shown in Figure 1. The plant protoplast transformation test showed that *AtARF1*, *2*, *3*, *4*, and *9* were the genes that inhibited gene expression [8,30]. AtARF1 contained amino acids rich in proline and the intermediate region of serine and threonine. A detailed sequence analysis of all 20 deduced FtARF proteins revealed that FtARF1, 2, 3, 4, 9, 10, 11, 13, 15, 16, 17, 18, and 23 contained the intermediate regions of proline, serine, and threonine, suggesting that these genes might have inhibitory effects (Figure S1). AtARF5, 6, 7, 8, and 19 were activators, and the middle was rich in glutamine [31,32]. Glutamine-rich regions were found in FtARF5, 6, 7, 8, 19, 20, and 21, indicating that these genes may be transcriptional activators during buckwheat development (Figure S1).

To explore the phylogenetic relationship of the FtARF proteins, a phylogenetic tree consisting of Arabidopsis (13 genes), rice (6 genes), maize (1 gene), and tartary buckwheat (20 genes) was constructed. The phylogenetic distribution showed that the *ARF* genes were divided into four major categories, category I, II, III, and IV, with well-supported bootstrap values (Figure 1a). Fifteen members were grouped in category I (6 members from tartary buckwheat), 7 members were grouped in category II (4 members from tartary buckwheat), 11 members were grouped in category III (6 members from tartary buckwheat), and 8 members were grouped in category IV (4 members from tartary buckwheat) (Figure 1a).

### 2.3. Gene Structure and Motif Composition of the FtARFs Gene Family

**Figure 2:**
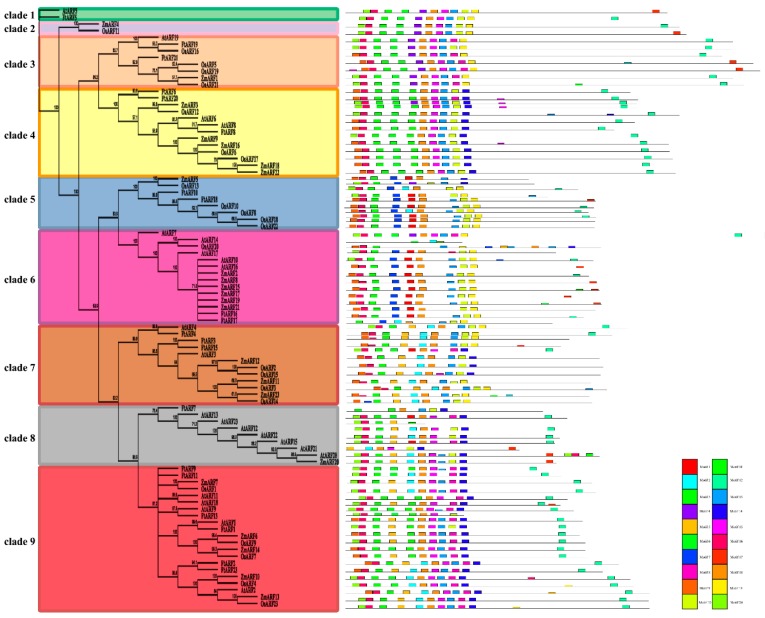
Phylogenetic relationships and motif compositions of ARF proteins from four different plant species. Left panel: an unrooted phylogenetic tree constructed using Geneious R11 with the neighbor-joining method. Right panel: distribution of conserved motifs in ARF proteins. The different-colored boxes represent different motifs and their position in each ARF protein sequence.

To understand the structural components of the *FtARF* genes, the exon and intron structures of the *FtARF* genes were obtained by comparison with the corresponding genomic DNA sequences (Figure 1c). The coding sequence of the entire FtARF family was disrupted by introns with exons ranging from 1 to 26 (Figure 1c). No gene with only one exon was observed. In general, the closest members from the same subfamily have similar exon/intron structures in terms of intron number and exon length. Further analysis showed that most FtARF proteins contained three characteristic regions (Figure 1d). The N-terminal region of all the FtARF proteins has a highly conserved region of approximately 100 amino acid residues corresponding to the DNA binding region. It is reported that the intermediate region of ARFs activates or inhibits domains and C-terminal AUX/IAA domains [30]. To further study the characteristic region of the FtARF proteins, the motifs of 20 FtARF proteins were analyzed using online MEME. Based on the results of MEME motif analysis, a schematic diagram was constructed to characterize the structure of the FtARF proteins. As shown in Figure 2d, with the exception of the division of the three domains of FtARF protein into 11 motifs, the members of FtARF in the same group usually have similar motifs. The ARF domain consisted of motifs 3, 5, and 10. Motif 1 constituted the DBD. The CTD corresponded to motifs 4, 8, 12, and 13. Motifs 1, 3, and 5 were found in 20 FtARF proteins. The similar motif of the FtARF protein in the subclass indicates that the protein structure is conserved in a specific subfamily, and the function of these conserved motifs remains to be elucidated. The conserved motif composition and similar gene structure of the same group of ARF members, coupled with the results of phylogenetic analysis, can support the reliability of population classification.

### 2.4. Evolutionary Analysis of the FtARF Genes and Several Different Species

**Figure 3:**
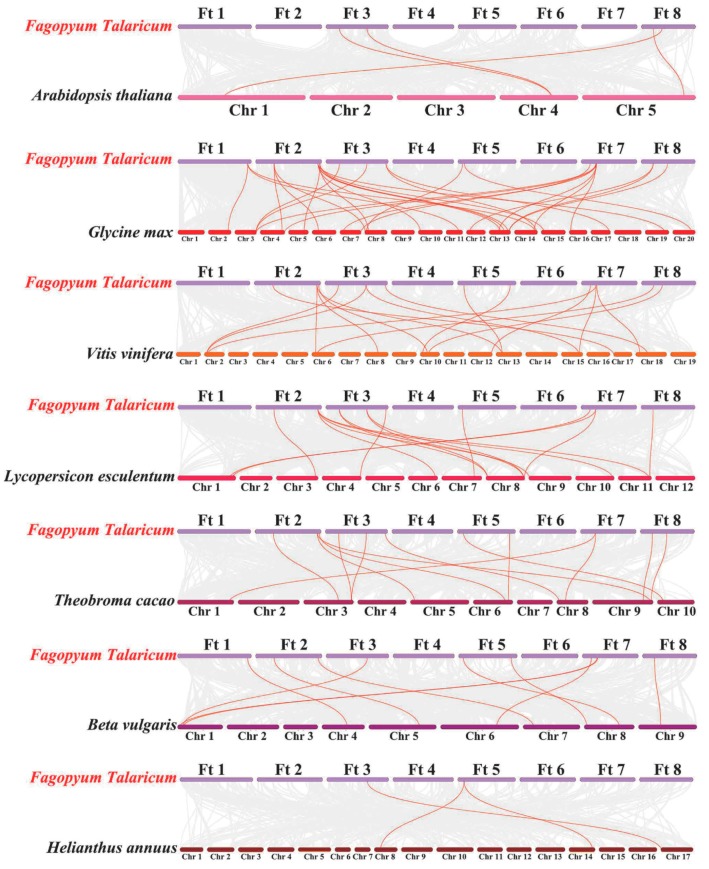
Synteny analysis of *ARF* genes between tartary buckwheat and seven representative plant species. Gray lines in the background indicate the collinear blocks within tartary buckwheat and other plant genomes, while the red lines highlight the syntenic *ARF* gene pairs.

The number of *FtARF* genes identified was similar to those of Arabidopsis thaliana, rice, and maize species *ARF* gene family members [13,14,33], but the genome size of the four species was very different (tartary buckwheat, 516 Mb; Arabidopsis thaliana, 125 Mb; rice, 450 Mb; maize, 2300 Mb). This indicated that the *ARF* gene family remained stable in different species over the long evolutionary process. Based on the existing *FtARF* gene, we studied the duplication and diversification of *ARF* genes in the course of evolution in more detail. The phylogenetic tree of the ARF protein sequence of two monocotyledonous plants (rice and maize) and two dicotyledonous plants (Arabidopsis thaliana and tartary buckwheat) was constructed by using Geneious R11 [33,34]. The ARF proteins were divided into nine clades by the phylogenetic tree as shown in Figure 3. Each of the four species contributed at least one *ARF* gene to Clade 3, Clade 4, Clade 6, Clade 7, and Clade 9, and the two branches (including Clade 3, 4 and Clade 5, 6, 7, 8, 9) were divided into several subbranches. ARF members from related species were clustered in these two branches. 

We also used MEME web servers to search for conservative motifs shared with the ARF proteins. Twenty different conserved motifs were found, and motifs 2, 3, 4, and 6 encode ARF domains. As shown in Figure 3, most ARF members in the same clade, especially the most closely related members, usually share common motifs (for example, FtARF8 and AtARF8), indicating potential functional similarities between the ARF proteins.

### 2.5. Chromosomal Distribution and Synteny Analysis of the FtARF Genes

The *FtARF* genes were unevenly distributed in 8 tartary buckwheat linkage groups (LG) (Figure 1b). Some linkage groups had more *FtARF* genes (LG3/8); LG3 had the most *FtARF* genes (5), and LG4/6 had no *FtARF* gene. Chromosomal regions within the 200 kb range of two or more genes are defined as tandem duplication events [35]. Six *FtARF* genes were clustered into three tandem repeat event regions in the tartary buckwheat linkage groups 2, 3, 5, and 8. LG3 had two clusters, indicating the hot spots of *FtARF* gene distribution. These results suggest that some *FtARF* genes may be produced by gene duplication, and these duplication events were the primary driving force of the evolution of FtARF. 

To further infer the phylogenetic mechanism of the FtARF family, we constructed seven representative comparative systematic maps of tartary buckwheat, including six dicots (Arabidopsis thaliana, soybean, grape, tomato, cocoa, and beet) and a monocot (sunflower) (Figure 3). A total of 14 *FtARF* genes showed a syntenic relationship with those in soybean, followed by grape (13), cocoa (12), beet (10), tomato (9), Arabidopsis (4), and sunflower (2). The homologous pairing numbers of the other six species (soybean, grape, cocoa, tomato, beet, Arabidopsis thaliana, and sunflower) were 27, 12, 11, 12, 9, 3, and 3, respectively. Some *FtARF* genes were associated with at least three syntenic gene pairs, especially tartary buckwheat and soybean *ARF* genes, such as *FtARF6* and *FtARF16*, which may play an important role in the evolution of the *ARF* gene family. Significantly, some *ARF* colinear gene pairs found between tartary buckwheat and soybean were anchored on highly conserved syntenic blocks, and the gene fragment spanned more than 100 genes. The relationship between tartary buckwheat and grape, cocoa, tomato, beet, and sunflower were similar, which may be related to the phylogenetic relationship between tartary buckwheat and the other six species. To better understand the evolutionary constraints of the *ARF* gene family, the Ka/Ks ratios of the *ARF* gene pairs were calculated. The Ka/Ks of most homologous ARF gene pairs were less than 1, indicating that the *FtARF* gene family may have experienced strong purification and selection pressure during the evolution of the *FtARF* gene family (Table S3).

### 2.6. Expression Patterns of the FtARF Genes in Different Plant Tissues

**Figure 4:**
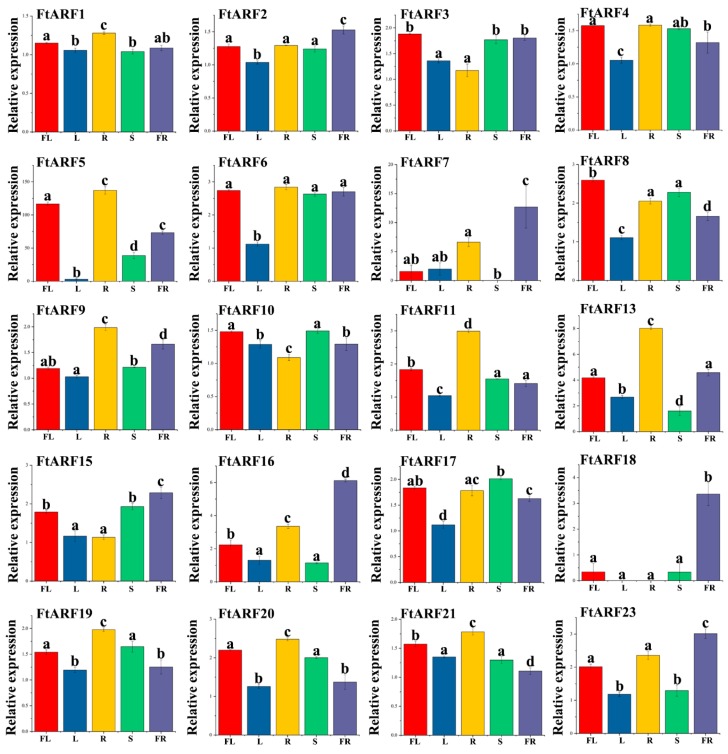
Expression profiles of 20 *FtARF* genes. The expression patterns of 20 *FtARF* genes in flower (FL), leaf (L), root (R), stem (S), and fruit (FR) tissues were examined by a qPCR assay. Error bars were obtained from three measurements. Small letter(s) above the bars indicate significant differences (α = 0.05, LSD) among the treatments.

**Figure 5:**
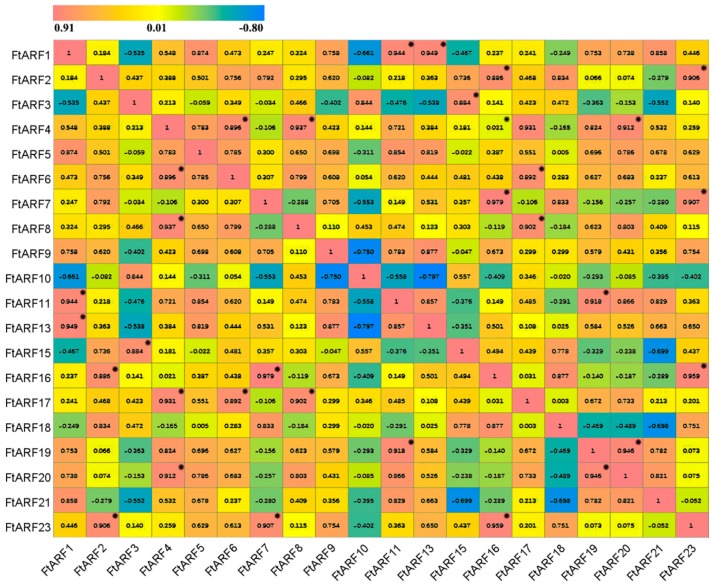
The correlations between the gene expression of the FtARFs in flower, leaf, root, stem, and fruit tissues. Red: positively correlated; blue: negatively correlated. * indicates significant correlation at 0.05 levels.

To investigate the physiological roles of the *FtARF* genes, the real-time PCR technique was used to detect the spatial expression of individual members of the gene family. The accumulation of the transcriptional products of 20 *FtARF* genes in the root, stem, leaf, flower, fruit, and other tissues were evaluated (Figure 4). The results showed that the transcriptional abundance of the *FtARF* genes varied greatly in different tissues and organs, suggesting that the *FtARF* genes had multiple functions in tartary buckwheat growth and development. Some *FtARF* genes showed organ/tissue-specific expression patterns. Six *FtARF* genes (*FtARF2/7/15/16/18/23*) were highly expressed in buckwheat fruit (Figure 4). Four *FtARF* genes (*FtARF3/4/8/10*) were expressed more in flowers than other organs. This study found that most of the *FtARF* genes, except *FtARF7/18*, were highly expressed in reproductive organs (Figure 4). At the same time, we studied the correlation of the *FtARF* gene expression pattern in roots, stems, flowers, leaves, and fruits (Figure 5). Most of the *FtARF* genes were positively correlated, and the *FtARF* genes (*FtARF1* and *FtARF11/13*; *FtARF2* and *FtARF16/23*; *FtARF3* and *FtARF15*; *FtARF4* and *FtARF6/8/17/20*; *FtARF6* and *FtARF17*; *FtARF7* and *FtARF17/23*; *FtARF8* and *FtARF17*; *FtARF11* and *FtARF19*; and *FtARF19* and *FtARF20*) that were significantly correlated were found to be positively correlated (Figure 5).

**Figure 6:**
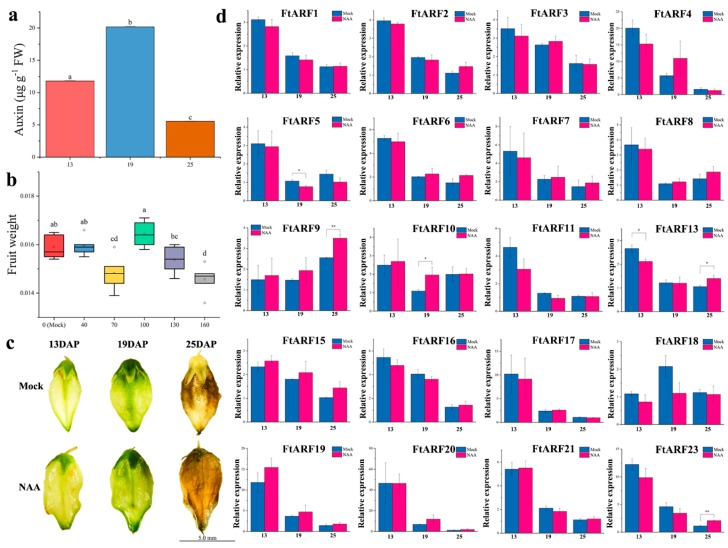
Fruit development of tartary buckwheat under exogenous auxin treatment. (**a**) IAA content during fruit development. (**b**) Final weight of the fruits treated with different concentrations of exogenous auxin. x-axis: weight of mature fruit, y-axis: concentration of naphthalene acetic acid treatment. (**c**) Pictures of the fruits treated with exogenous auxin during fruit development. (**d**) Differences in the expression of 20 *FtARF* genes under exogenous auxin treatment during fruit development. Mock: the same amount of water treatment, NAA: 100 mg·L^−1^ naphthalene acetic acid treatment. Error bars were obtained from three measurements. Small letter(s) above the bars indicate significant differences (α = 0.05, LSD) among the treatments. * and ** indicate significant correlation at 0.05 and 0.01 levels, respectively.

**Figure 7:**
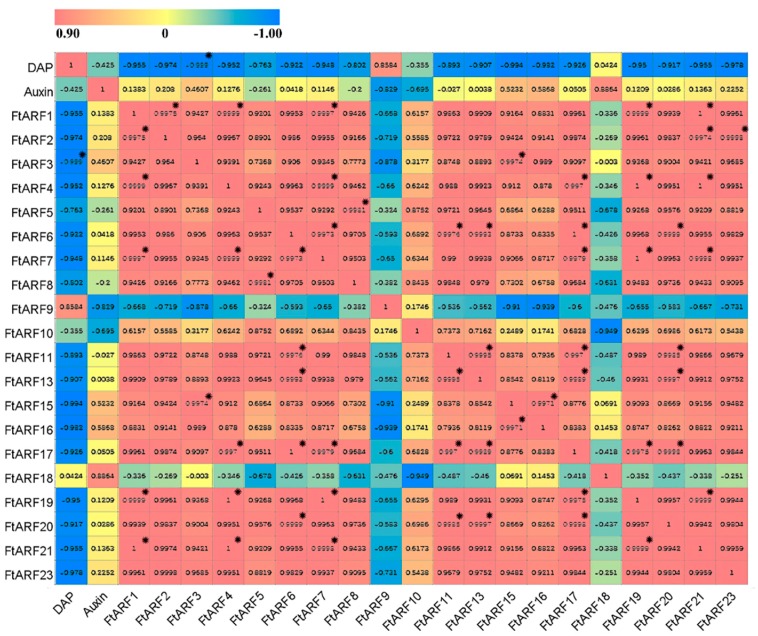
The correlation between the gene expression of FtARFs and auxin during fruit development. Red: positively correlated; Blue: negatively correlated. * indicates significant correlation at 0.05 levels.

First, we measured the content of endogenous auxin during the development of tartary buckwheat fruits. It was found that the content of endogenous auxin reached its maximum at 19 days after pollination (DAP). The content of endogenous auxin in early development (13 DAP) was higher than that in late development (25 DAP) (Figure 6a). Most of the *FtARF* genes were expressed during the process of fruit development (13, 19, and 25 DAP). In general, most of the FtARF were higher in the early stage of fruit development than in the later stages of fruit development (Figure 6d). The expression level of the 13 DAP *FtARF* genes in the fruit were higher than that in the other stages, while the expression level of two *FtARF* genes (*FtARF9/18*) in 13 DAP were lower than that in other stages, and three *FtARF* genes (*FtARF17/19/20*) showed a high transcription level in 19 DAP (Figure 6d). Further analysis showed that with the fruit enlargement (Figure 6c), most of the *FtARF* genes were negatively correlated (except FtARF9/18), especially the *FtARF3* gene. Most of the *FtARF* genes were positively correlated with the endogenous auxin content (except FtARF5/8/9/10). Most of the *FtARF* genes were positively correlated, especially the *FtARF* genes (*FtARF1* and *FtARF2/4/7/19/21*; *FtARF3* and *FtARF15*; and *FtARF4* and *FtARF7/17/19/21*). We also found that the *FtARF9/18* gene was negatively correlated with most other *FtARF* genes during the development of fruit (Figure 7).

### 2.7. Differential Expression of the FtARF Genes during Fruit Development under NAA Treatment Conditions

To further determine the response of the *FtARF* genes to auxin, different concentrations of NAA (40, 70, 100, 130, or 160 mg·L^−1^) were sprayed on the whole tartary buckwheat plants in the bud stage. The fresh weight of the mature fruit increased significantly to 0.0164 g following treatment with 100 mg·L^−1^ NAA (Figure 6b). In Figure 6c, 100 mg·L^−1^ NAA was the best choice for increasing fruit weight. NAA with concentration greater than or less than 100 mg·L^−1^ had no significant effect on the weight gain of fruit; even too high concentration would reduce the weight of fruit (Figure 6c). Therefore, we studied the expression of *FtAFR* genes in more detail by exogenously spraying them with NAA.

We found that the expression of the *FtARF* gene changed with the exogenous NAA. In the early stage of development (13 DAP), the expression of most of the *FtARF* genes was lower than that of the mock group (except *FtARF9/10/15/19/20/21*). In the late stage of development (25 DAP), the expression of most *FtARF* genes was higher than that of the mock group (except *FtARF4/5/11/13/17/18*). The whole development period of tartary buckwheat fruit was observed. When exogenous NAA was applied, the expression pattern of all the *FtARF* genes remained the same. For example, the expression of *FtARF5* in 19 DAP was significantly decreased; the expression of *FtARF10* in 19DAP was significantly increased; *FtARF13* significantly decreased/increased in 13/25DAP, and the expression of *FtARF23* in 25 DAP was significantly increased. The results showed that the *FtARF* genes could respond positively to auxin during the fruit development (Figure 7).

## 3. Discussion

*ARF* genes are a class of transcription factor family. The *ARF* gene family, which exists comprehensively in all plant species, has been widely used in many species that have been sequenced [34,36,37]. In this study, a search for *ARF* genes in the tartary buckwheat genome resulted in the identification of 20 members, which were designated *FtARF1* through *FtARF23* on the basis of their chromosomal location, with the exception of *FtARF12/14/22*.

The conserved domain of the ARF protein in tartary buckwheat was studied. Multiple sequence alignments showed that the ARF domain of five FtARF proteins (FtARF8, FtARF10, FtARF16, FtARF17, and FtARF18) had sequence variation. Based on previous studies, the ARF domain located between DBD and CTD may affect the normal interaction between the *ARF* genes and downstream target genes, based on its amino acid composition and the transcriptional activation or inhibition of the change in the ARF domain motif. Therefore, these five ARF proteins may merit further study of their function and binding specificity [8,30].

Multiple members of the specific gene family of a particular organism are thought to be natural products of the long evolutionary process of the organism. The number of members of a gene family reflects the recombination and expansion of the genome due to the extensive duplication and diversity that frequently occurs during evolution. The comparison of *FtARF* genes with those in other dicotyledonous genomes showed that the number of tartary buckwheat genes was relatively small [38]. Tandem and segmental duplication events play an important role in the expansion of *ARF* genes [39]. Genomic duplication events are common in angiosperm evolution and usually lead to the expansion of the gene family [40]. Several rounds of whole genome duplication have been found in Arabidopsis thaliana and buckwheat genomes and reported in previous studies [41,42,43]. However, the σ genome duplication (WGD) event was shared with all Poales, while the ρWGD event is hypothesized to have occurred after different lineages led to the occurrence of the Gramineae plants and buckwheat in Polygonaceae plants [40]. Therefore, the absence of the pan-grass ρWGD event during the evolution of tartary buckwheat may be one of the reasons for the low number of *FtARF* genes in buckwheat.

The presence of the duplicated *FtARF* genes raises the issue of their functional redundancy. According to the evolutionary model, duplicated genes may undergo different processes of selection: a copy is dysfunctionalized by the loss of function, a copy of the expression/function decline of the low functionalization, and a copy of the new function of the new functionalization. Another possibility is that two copies are partitioned or specialized into the subfunctionalization of different functions. These evolutionary fates may lead to differences in expression patterns or protein structures. Our study found that most duplicated FtARFs were expressed in different tissues/organs, suggesting that these genes had specific or redundant cellular functions [41,43,44]. The evidence of differences between duplicate genes can be deduced from the expression patterns of the *FtARF7* and *FtARF13* genes. *FtARF13* was highly expressed in the stems, but the *FtARF7* gene was not expressed. In addition, possible subfunctionalization changes the expression pattern of the gene pairs. For example, the mRNA abundance of FtARF3 peaked in the flower, but FtARF15 was highly expressed in the seed.

Fruit development is a complex interaction of cell division, differentiation, and expansion, which occurs in a spatiotemporal coordinated manner in reproductive organs [45]. Auxin triggers and/or promotes the division and elongation of unpollinated stationary ovaries, which is considered to play an important role in fruit formation and development [46,47]. Auxin signaling plays an important role in embryonic development. For example, higher levels of auxin were detected in the cotyledon protoplasts of Arabidopsis thaliana from the heart to the root tips and the ends of mature embryos [48]. SlARFs are involved in the various types of regulation of fruit development in tomato [49]. SlARF7 plays a negative role in fruit setting after pollination and fertilization and regulates the response of auxin during fruit growth [50]. Another tomato gene, *SlARF4*, is an auxin response factor involved in the control of glucose metabolism during fruit development and is expressed in the pericarp of immature fruit [24]. During the fruit development, a negative correlation was found between the expression of the *FtARF* genes and the development of fruit (except *FtARF9/18*), in particular, *FtARF3*. The expression pattern of auxin in the buckwheat fruit is consistent with that of *FtARF18* during fruit development. 

Since the ARFs are a group of transcription factors that regulate the expression of the auxin response genes, it is highly significant to determine the response of the *FtARF* genes to auxin treatment. It was reported that the transcripts of Arabidopsis ARF4/5/16/19 and rice OsARF1/23 were slightly increased by auxin, while those of OsARF5/14/21 decreased slightly [13,14,51,52,53]. In this study, we found that most of the *FtARF* genes were responsive to exogenous auxin treatment, and *FtARF5/10* had a significant change in 19 DAP. *FtARF9/13/23* was upregulated significantly at 25 DAP. It is hypothesized that these *FtARF* genes are more responsive to auxin during buckwheat fruit development.

In summary, these findings provide insight into the potential function of *FtARF* genes. Comprehensive analysis was helpful to screen *ARF* genes for further functional identification and the genetic improvement of agronomic traits of tartary buckwheat.

## 4. Materials and Methods

### 4.1. Gene Identification and Classification

The tartary buckwheat genome was downloaded from the Tartary Buckwheat Genome Project (TBGP; Available online: http://www.mbkbase.org/Pinku1/). The *FtARF* gene family members were identified by a BLASTp search. The *FtARF* gene was searched by two BLASTp methods, and the maximum number of *ARF* genes was determined. First, all known *Arabidopsis ARF* genes were used to query the initial protein on the TBGP website, and the candidate genes were identified by a BLASTp search at a score value of ≥100 and e-value ≤ 1 × 10^−10^ [1]; when there was no result, *Zea mays* or *Oryza sativa ARF* genes were used. Second, the hidden Markov model (HMM) file corresponding to the ARF domain (PF06507) was downloaded from the Pfam protein family database (Available online: http://pfam.sanger.ac.uk/). The *ARF* genes were retrieved from the tartary buckwheat genomic database with HMMER3.0. The default parameter cutoff was set to 0.01. The existence of the ARF core sequences was verified with the PFAM and SMART programs, and the HMMER results of all the candidate genes that might contain the ARF domain were further verified. Finally, 20 *ARF* gene models were identified in the tartary buckwheat genome for further analysis. The sequence length, molecular weight, isoelectric point, and subcellular localization of the ARF protein identified were obtained using the tools of the ExPasy website (Available online: http://web.expasy.org/protparam/).

### 4.2. Sequence Analysis

To study the structural differences between the *FtARF* genes, conserved motifs were studied in the ARF proteins encoded. Using the ARF domain sequence of the FtARF proteins, we used the default parameter ClustalW to compare several protein sequences. The predicted coding sequence was compared with the corresponding full-length sequence using the Gene Structure Display Server (GSDS; Available online: http://gsds.cbi.pku.edu.cn) online program, and the exon–intron structure of the *FtARFs* genes was determined. To determine the conserved motifs of the FtARFs proteins recognized, a MEME online program (Available online: http://meme-suite.org/tools/meme) was used to analyze the protein sequences with the following parameters: the optimum motif width was 6~200, and the maximum number of motifs was 20 [54].

### 4.3. Chromosomal Distribution and Gene Duplication of FtARF Genes

All the *FtARFs* genes were mapped to the chromosomes from the physical location information obtained from the tartary buckwheat genomic database using Circos [55]. Multiple collinear scanning toolkits (MCScanX) were used to analyze gene duplication events with the default parameters [56]. To reveal the synteny relationship of orthologous *ARF* genes between tcartary buckwheat and other species selected, the syntenic analysis maps were constructed using the Dual Systeny Plotter software (Available online: https://github.com/CJ-Chen/TBtools) [57]. The substitution of nonsynonymous (Ka) and synonymous (Ks) for each repeated *ARF* gene were calculated using the KaKs_Calculator 2.0 [58].

### 4.4. Phylogenetic Analysis and Classification of the FtARF Gene Family

Based on the classification scheme of AtARF and the ARF domain of FtARF and AtARF, all the *FtARF* genes identified were divided into different groups. Phylogenetic trees were constructed using ARF protein sequences (Arabidopsis thaliana, maize, rice, and soybean) downloaded from the UniProt database (Available online: https:/www.uniprot.org). The phylogenetic trees were derived by using the neighbor-joining (NJ) method of Geneious R11. The parameters were Blosum62 cost matrix, and Jukes–Cantor model, global alignment with free end gaps.

### 4.5. Plant Growth and Treatments

Tartary buckwheat accessions used in this study (MIQIAO) were requested from Professor Wang Anhu of Xichang University. MIQIAO is a dehulled variety of tartary buckwheat obtained by physical and chemical mutagenesis [59]. From 2013 to 2018, MIQIAO was introduced into the experimental field of the College of Life Science, Sichuan Agricultural University (Lat. 29°97′ N, 102°97′ E, Alt. 580 m), Ya’an, Sichuan, China, and grown in the same ecological environment and cultivation conditions. The materials were collected in 2017. The flower from the flowering stage, the fruit from three (13, 19, and 25 DAP) different developmental stages of fruit, the stem, root, and leaf of mature tartary buckwheat were collected separately for RNA extraction and used for further qRT-PCR analysis. The samples were flash frozen in liquid nitrogen and stored at −80 °C for further use.

In the bud stage, tartary buckwheat with a similar growth state were sprayed once with 40, 70, 100, 130, or 160 mg·L^−1^ of naphthalene acetic acid (NAA), and the same amount of water was sprayed as the control (Mock). When fully ripe, the weight of the fruits was measured. At 13 DAP (green fruit stage), 19 DAP (expansion stage), and 25 DAP (discoloration stage) [60], the fruit samples were rapidly frozen in liquid nitrogen and stored at −80 °C for further use.

### 4.6. Phytohormone Analysis

Samples of fresh material that weighed approximately 0.5 g were ground in liquid nitrogen. The resulting powders were homogenized in 10 mL of 80% methanol and then stirred overnight at 4 °C. Subsequently, each suspension was centrifuged at 13,900× *g* for 10 min under refrigeration (4 °C). The supernatant was collected and 5 mL of 80% methanol was added to the residue. Again, the supernatant was collected after centrifugation. The pooled supernatant (~15 mL) was flash evaporated at 36 °C until there was no methanol (~3 mL). Then, the rotary evaporator bottle was washed with 5 mL of ultrapure water. Then, the rinse was combined with the residual liquid (~3 mL). The solution was decolorized with 15 mL of diethyl ether three times, and the ether phase was discarded. The aqueous phase was collected and basified to pH 8.0 with 0.1 M Na_2_HPO_3_. The basified extract was placed in a shaker for 30 min with 50 mg of polyvinylpyrrolidone at 4 °C and centrifuged at 13,900× *g* for 10 min. The supernatant was collected and acidified to pH 3.0 with 0.2 M citric acid. The solution was separated three times with 5 mL of ethylacetate, and the aqueous phase was discarded. The pooled ethylacetate phase (~15 mL) was flash evaporated at 36 °C to near dryness. The residue was dissolved in 1 mL of methanol [61,62,63].

Each sample was filtered through a nylon 66 filter (25 mm diameter, 0.45 μm pore size) prior to injection into an HPLC column. The HPLC analysis was performed on an Agilent 1260 system using a C18-ODS (3.5 µm × 150 mm × 4.6 mm) column (Agilent, Santa Clara, CA, USA) and a UV/VIS detector. An injection volume of 10 µL, a column temperature of 35 °C, a flow rate of 1 mL min^−1^, and a run time of 10 min were maintained for all analyses. The system was calibrated with external standards of indole-3-acetic acid (IAA). For detection, separation was performed with a mixture of methanol and distilled water containing 0.6% acetic acid (*V*:*V* = 50:50) and isocratic elution. The elutant was scanned at 257 nm.

### 4.7. Expression Analysis of the FtARF Genes Using Real-Time PCR

First, the corresponding sequences of these genes were obtained from the tartary buckwheat (Pinku1) genome sequence database (Available online: http://www.mbkbase.org/Pinku1/), and then the RT-qPCR primers were designed by Primer3 software (Available online: http://frodo.wi.mit.edu/) (Table S4). Finally, quantitative real-time PCR analysis was used to analyze the identified genes.

Because histone *H3* gene can be continuously expressed in almost all tissues and every growth stage of organism and is extremely conserved in evolution, it is often used as a reference gene. Using the *FtH3* gene as internal control, the standard RT-qPCR with SYBR Premix Ex Taq II (Tokyo, Japan, TaKaRa) was repeated at least three times on a CFX96 Real-Time System (Hercules, CA, USA, BioRad). The data were analyzed by the 2^−(∆∆*C*t)^ method, and the related mRNA expression data were obtained [64].

### 4.8. Statistical Analysis

All the data were analyzed by an analysis of variance using the Origin Pro 2018b (OriginLab Corporation., Northampton, MA, USA) statistics program, and the means were compared by the least significant difference test (LSD) at the 0.05 and 0.01 level of significance.

## Supplementary Materials

The following are available online at http://www.mdpi.com/1422-0067/19/11/3526/s1, Figure S1: Alignment of multiple FtARF and selected ARF domain amino acid sequences, Table S1: List of the 20 *FtARF* genes identified in this study, Table S2: Analysis and distribution of conserved motifs in tartary buckwheat ARF proteins, Table S3: One-to-one orthologous relationships between tartary buckwheat and other seven plant species, Table S4: Primers of sequences.

## Abbreviations

ABA	abscisic acid
ARF	auxin response factor
AUX	auxin
Aux/IAAs	auxin/indole acetic acid
CDS	coding sequence length
CTD	carboxyl-terminal dimer
DAP	days after pollination
DBD	N-terminal B3 DNA binding domain
GH3s	Gretchen Hagen 3
HPLC	high-performance liquid chromatography
IAA	indoleacetic acid
Ks	synonymous
Ka	nonsynonymous
LG	linkage groups
LSD	least significant difference test
MR	intermediate region
MW	molecular weight
NAA	naphthalene acetic acid
PI	isoelectric point
RT-qPCR	reverse transcription-quantitative PCR
SAURs	small auxin up RNAs
