# Supplementary figures and images for: Genome-Wide Investigation of the Auxin Response Factor Gene Family in Tartary Buckwheat (Fagopyrum tataricum)
Source: Int J Mol Sci. 2018 Nov 9;19(11):3526. doi: 10.3390/ijms19113526 (PMC6274889; doi:10.3390/ijms19113526)

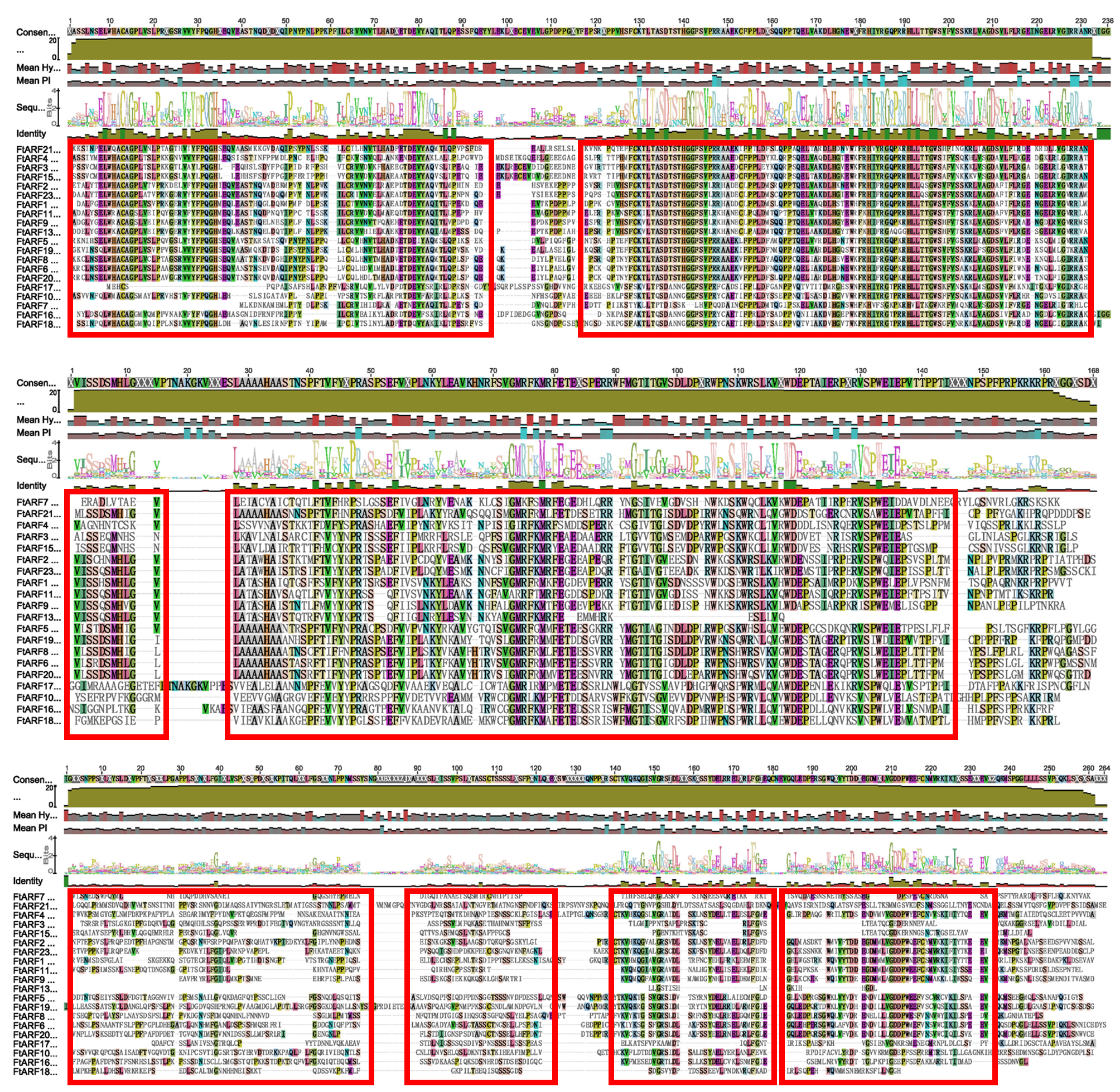

Supplement: Supplementary file 1 [file ijms-19-03526-s001.zip › ijms-383622-supplementary/Supplementary Figure 1.tif]
